# Point prevalence survey of antibiotic consumption across three hospitals in Ghana

**DOI:** 10.1093/jacamr/dlab008

**Published:** 2021-02-08

**Authors:** Obed Kwabena Offe Amponsah, Kwame Ohene Buabeng, Alex Owusu-Ofori, Nana Kwame Ayisi-Boateng, Katri Hämeen-Anttila, Hannes Enlund

**Affiliations:** 1 Department of Pharmacy Practice, Faculty of Pharmacy and Pharmaceutical Sciences, College of Health Sciences, Kwame Nkrumah University of Science and Technology, Kumasi, Ghana; 2 Department of Clinical Microbiology, Komfo Anokye Teaching Hospital, Kumasi, Ghana; 3 School of Medicine and Dentistry, College of Health Sciences, Kwame Nkrumah University of Science and Technology, Kumasi, Ghana; 4 University Hospital, University Health Services, Kwame Nkrumah University of Science and Technology, Kumasi, Ghana; 5 Department of Assessment of Pharmacotherapies, Finnish Medicines Agency, Kuopio, Finland

## Abstract

**Background:**

Actionable data on antimicrobial use is important when planning strategic interventions such as antimicrobial stewardship to address the challenge of drug resistance, particularly in resource-constrained settings.

**Objectives:**

To assess the prevalence of antibiotic use, the pattern of commonly used antibiotics and patient factors that may be associated with the increased use of antibiotics in the study hospitals.

**Methods:**

This was a cross-sectional study conducted using the WHO Methodology for Point Prevalence Surveys in hospitals. Chi-squared analysis, Fisher’s exact test and logistic regression were employed to analyse statistically the data obtained.

**Results:**

The overall prevalence of antibiotic use in the hospitals was 60.5%. The commonest indications for antibiotic recommendations were community-acquired infections (36.5%), surgical prophylaxis (26.1%) and hospital-acquired infections (15.7%), among others. Very few (2.7%) of the patients had their samples taken for culture and susceptibility testing to guide therapy. Penicillins (48.7%), cephalosporins (23.5%) and fluoroquinolones (17.4%) were the most commonly prescribed antibiotics. Concurrent malaria infection [adjusted OR (AOR) 0.33, 95% CI 0.11–0.94, *P = *0.04] and increasing age (AOR 0.98, 95% CI 0.96–1.00, *P = *0.02) were associated with lower risk of antibiotic use.

**Conclusions:**

The prevalence of antibiotic consumption in the hospitals was lower than that reported in similar studies in Ghana, but high relative to some reports from high-income countries. Most antibiotic therapy was empirical and not guided by culture and susceptibility testing. There is the need for application of the WHO *AWaRe* classification for the selection of antibiotics and increased use of culture and susceptibility data to guide infectious disease therapy.

## Introduction

Antimicrobial resistance (AMR) is an important subject in healthcare and public health as it is associated with increased mortality and morbidity among patients. The risk of AMR is high as every individual is susceptible to developing drug-resistant infections.^1^ Infections due to drug-resistant organisms require more aggressive therapy to be used, including (but not limited to) the use of combinations of different agents and the use of reserved agents. Treatment of resistant infections is associated with higher costs^2^ for second line drugs, additional investigations, and longer hospitalization.^3^ Productivity losses due to excess morbidity and premature mortality are related indirect costs posing profound socioeconomic problems and implications for the world.^4^ WHO has recognized AMR as an important public health threat, requiring immediate intervention to slow its progression around the world.^5^ The WHO, in a collaborative effort, has already developed the Global Action Plan (GAP) using the One Health approach to fight AMR in veterinary and human medicine.^6^ With this, countries around the world are expected to tailor interventions and mechanisms to tackle AMR to their local circumstances.

Data and information on the use of antimicrobials in relevant sectors are indispensable in policy-making to tackle AMR. Information-driven solutions make the most sustainable impact with minimal resources and wastage. Real world data is needed to implement locally tailored antimicrobial stewardship programmes (ASPs) to optimize antibiotic use. WHO develops various tools and strategies to support stewardship activities in countries.^6^ One of these is the 2019 *AWaRe* Classification database, first developed in 2017, which has 180 antibiotics classified as *A*ccess, *Wa*tch or *Re*serve (*AWaRe*), based on pharmacological class, anatomical therapeutic chemical (ATC) codes and their WHO Essential Medicines List status. This classification provides a useful means to monitor antibiotic use across the classes as an indicator of optimal antibiotic use. Antibiotics in the *Access* group are those that show a lower potential for resistance than the other groups with a good range of activity against many common pathogens. *Watch* antibiotics are those with much higher resistance potential and as such require key targets for stewardship interventions to reduce their inappropriate use. The *Reserve* group are those used for treating infections due to multidrug-resistant pathogens and so should be used as a last line, after all alternatives have been explored, in order to protect them against resistance.^7^

Several studies have investigated antibiotic use in various settings in Ghana and across the world with varied prevalence of antibiotic use and patient parameters affecting their use.^8–16^ However, impactful interventions that optimize antimicrobial use require facility-specific data to determine which specific improvements can be made. The current study investigated antibiotic use in three hospitals in the Ashanti region to gather data for implementation of antimicrobial stewardship. We aimed to determine prevalence of antibiotic use, commonly used antibiotics, patient variables associated with increased antibiotic use in the hospitals and to identify targets for interventions by ASPs.

## Methods

### Study design and setting

We employed the WHO Methodology for Point Prevalence survey (PPS) on antibiotic use in hospitals, version 1.1, which has been described elsewhere.^17^

The facilities were classified according to the criteria set forth in the WHO methodology for PPS^17^ used for the study. Kwame Nkrumah University of Science and Technology (KNUST) Hospital (UHS) was classified as tertiary, Agogo Presbyterian Hospital (APH) as secondary and Ejisu Government hospital (EGH) as a primary facility.

UHS provides services to more than 200 000 Ghanaians annually. The hospital provides a wide range of services ranging from ambulatory to in-patient care. The hospital has a functioning infection prevention and control programme as well as a drug and therapeutics committee supporting optimization of patient care.^18^

APH, the secondary hospital, is a 250 bed capacity hospital which caters to patients from all over Ghana and neighbouring countries, especially for ophthalmological care. It serves a municipal catchment area of over 170 822 persons. It is designated as Collaborating Centre for the University of Ghana School of Public Health, Training Centre for Buruli Ulcer Treatment, Ministry of Health (MOH)/WHO designated centre for training in the surgical management of buruli ulcer and one of two sites in Ghana and eight sites in Africa for Malaria Vaccine Trial.^19^

EGH is a primary hospital facility under the Ghana Health service in a municipality of over 143 762 persons.^20^ The hospital is in the capital of the municipality which is a bustling market city that sees many people coming in, especially on market days (Thursdays and Sundays).

### Point prevalence survey

The PPS was a cross-sectional survey conducted on 26 and 27 November 2019 at a tertiary hospital (UHS) and primary level facility (EGH), respectively and on 10 December 2019 at a secondary level hospital (APH). The different dates were necessary to ensure that all in-patients at each facility were surveyed with the resources available.

Each patient present on the ward at the time of the survey was targeted for inclusion in the study unless the patient did not meet inclusion criteria. Generally, any form with more than 10% missing data would be excluded from the analysis. Table 1 shows the inclusion and exclusion criteria.

**Table 1.** Patient inclusion and exclusion criteria for point prevalence survey

Level	Inclusion	Exclusion
Ward	All acute care inpatient wards in the hospitals.	Long-term care wards, Emergency departments (except for wards attached to be monitored for more than 24 h), Day surgery and Day care wards.
Patients	Hospitalized patient already admitted in the ward as at 08:00 am on day of survey whether receiving antibiotic treatment or not.	Patients admitted after 08:00 am on day of survey, all Day care patients.
Antibiotics	Only antibiotics included in ANNEX XI of the WHO protocol administered by oral, parenteral, rectal or inhalation routes were included, antibiotic initiated by 08:00 am on survey day.	Topical antibiotics, antibiotic started after 08:00 am on day of survey.

### Data collection

Anonymized data was collected from the hospital records of in-patients who met the inclusion criteria. The data was collected by the research team with help from select final year Doctor of Pharmacy students from KNUST. A training programme was organized by the research team led by O.K.O.A. and N.K.A.B. on the 25 November 2019 for UHS and EGH as well as on 9 December 2019 for APH, to ensure accuracy and minimize error during data collection. This training involved simulations on obtaining information from patient records, and managing situations and records that strayed from the norm. A mock data collection exercise was conducted to improve accuracy during data collection. A total of 16 persons collected patient data at UHS and EGH and 14 persons at APH. All data was collected on hard copies of forms used in the protocol. No patient sampling was done.

### Data management and analyses

Data collected after surveying each hospital was sorted and organized to prevent mix-up during data entry. All the data collected in the study was entered into a REDCap^®^ database and exported into Stata™ 14 for analyses.^21^^,^^22^ No data forms had more than 10% missing data recorded and so all forms were included in the entry and subsequent analyses. Missing data were entered as ‘unknown’ in the database. Descriptive analysis with frequencies and percentages show the patterns within variables. Chi-squared tests and or Fisher’s exact test where appropriate was used to test univariate associations between antibiotics use and patient variables collected in the survey. Finally, forward stepwise logistic regression model with unadjusted and adjusted odds ratios was constructed to establish whether there is a relationship between the antibiotics use and surgery on admission, co-morbidities, nutritional status, use of catheter, tuberculosis and HIV status, peripheral vascular catheter, gender, age and type of infection. A *P* value of <0.05 was considered statistically significant.

### Ethics

Ethics approval was obtained from the KNUST Committee on Human Research, Publications and Ethics after getting approval from each facility. (CHRPE/AP/654/19).

## Results

190 out of 211 inpatients met the inclusion criteria for the study: 44 out of 49 from UHS, 45 of 49 from EGH and 101 of 113 from APH.

The overall prevalence of antibiotic use was 60.5% (Table 2). APH, the secondary hospital, had the highest prevalence at 67.3% while the primary hospital EGH had the lowest prevalence at 51.1%.

**Table 2.** Demographics and prevalence of antibiotic use by hospital

Factor	*n* (%) or median (IQR)			
	Overall (*N = *190)	UHS (*n = *44)	APH (*n = *101)	EGH (*n = *45)
Gender				
Female	120 (63.2%)	29 (65.9%)	60 (59.4%)	31 (68.9%)
Male	63 (33.2%)	13 (29.6%)	36 (35.6%)	14 (31.1%)
Age				
≥2 years (*N = *165)	30 (21–48)	27.5 (21.0–42.0)	35.0 (20.0–57.0)	29.0 (21.0–44.0)
0–23 months (*N = *21)	0.13 (0.06–0.30)	0.03 (0.03–0.13)	0.2 (0.1–0.3)	0 (0.0–7.0)
Patients on antibiotics	115 (60.5%)	68 (67.3%)	24 (54.5%)	23 (51.1%)
Number of antibiotics per patient (*N = *115)				
1	37 (32.2%)	26 (38.2%)	4 (16.7%)	7 (30.4%)
2	68 (59.1%)	34 (50.0%)	19 (79.2%)	15 (65.2%)
3	10 (8.7%)		1 (4.2%)	1 (4.4%)

*N*=Total number of patients; *n*=number of patients.

Table 3 describes the distribution of the indications for use of antibiotics in the facilities and if such therapy had a culture sample taken or not. Most antibiotics were used for community-acquired infections (36.5%), surgical prophylaxis (26.1%) and for hospital-acquired infections (15.7%). Most prescriptions were empirical as only 2.7% of cases had a sample taken for culture and susceptibility analyses.

**Table 3.** Summary of antibiotic indication

		Hospital		
Factor (*n = *115)	Total	UHS	APH	EGH
Indication type				
Community-acquired infection	42 (36.5%)	10 (41.7%)	21 (30.9%)	11 (47.8%)
Surgical prophylaxis	30 (26.1%)	9 (37.5%)	17 (25.0%)	4 (17.4%)
Hospital-acquired infection	18 (15.7%)	1 (4.2%)	15 (22.1%)	2 (8.7%)
Medical prophylaxis^a^	16 (13.9%)	3 (12.5%)	7 (10.3%)	6 (26.1%)
Other	9 (7.8%)	1 (4.2%)	8 (11.8%)	0 (0.0%)
Reason for antibiotic use in notes?				
Yes	89 (88.1%)	23 (95.8%)	44 (80.0%)	22 (100.0%)
Culture sample taken?				
Yes	3 (2.7%)	2 (8.3%)	0 (0.0%)	1 (4.6%)

Results shown are *n* (%).

a Antibiotics given to prevent bacterial infections in susceptible groups, such as patients with Crohn’s disease, pregnant women with suspected/susceptible to preterm/premature rupture of membranes to prevent early-onset neonatal Group B streptococcal disease.^39^

**Figure 1.**
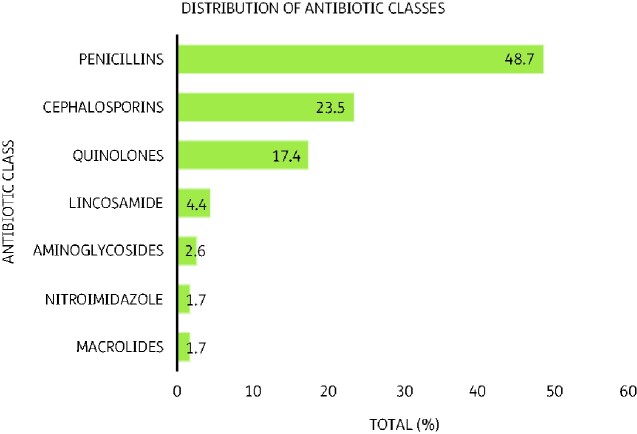
Prescribed antibiotics by antibiotic classes.

The commonly prescribed antibiotics were penicillins (48.7%) cephalosporins (23.5%) and fluoroquinolones (17.4%) as shown in Figure 1. Table 4 shows the commonly used agents as amoxicillin (36.5%), ciprofloxacin (17.4%), ceftriaxone (11.3%), cefuroxime (9.6%) and ampicillin (7.8%).

**Table 4.** Prescribed antibiotics by hospital

		Hospital		
Antibiotic^a^ (*AWaRe* class)	Overall	UHS	APH	EGH
Amoxicillin (*A*)	42 (36.5%)	12 (50.0%)	18 (26.5%)	12 (52.2%)
Ciprofloxacin (*W*)	20 (17.4%)	3 (12.5%)	15 (22.1%)	2 (8.7%)
Ceftriaxone (*W*)	13 (11.3%)	2 (8.3%)	11 (16.2%)	0 (0.0)
Cefuroxime (*W*)	11 (9.6%)	3 (12.5%)	6 (8.8%)	2 (8.7%)
Ampicillin (*A*)	9 (7.8%)	0 (0.0)	9 (26.5%)	0 (0.0)
Clindamycin (*A*)	5 (4.4%)	0 (0.0)	3 (4.4%)	2 (8.7%)
Benzylpenicillin (*A*)	3 (2.6%)	0 (0.0)	1 (1.5%)	2 (8.7%)
Amikacin (*A*)	2 (1.7%)	0 (0.0)	0 (0.0)	2 (8.7%)
Cefotaxime (*W*)	2 (1.7%)	0 (0.0)	2 (2.9%)	0 (0.0)
Flucloxacillin (*U*)	2 (1.7%)	0 (0.0)	2 (2.9%)	0 (0.0)
Metronidazole (*A*)	2 (1.7%)	0(0.0)	1 (1.5%)	1 (4.4%)
Azithromycin (*W*)	1 (0.9%)	1 (4.2%)	0 (0.0)	0 (0.0)
Cefpodoxime (*U*)	1 (0.9%)	1 (4.2%)	0 (0.0)	0 (0.0)
Erythromycin (*W*)	1 (0.9%)	1 (4.2%)	0 (0.0)	0 (0.0)
Gentamicin (*A*)	1 (0.9%)	1 (4.2%)	0 (0.0)	0 (0.0)

Results shown are *n* (%).

WHO *AWaRe* classification: *A*, Access (46.7%); *W*, Watch (40%); *Re*, Reserve (0); *U*, Unclassified (13.3%).

a Antibiotic names are the International Nonproprietary Name (INN).

According to the univariate analysis, surgery, urinary catheter and peripheral vascular catheter use all showed statistically significant association with antibiotic use (Table 5). Results for each individual hospital are shown in Tables S1 to S3 (available as Supplementary data at *JAC* Online).

**Table 5.** Patient variables and association with antibiotic use

	Patients on antibiotics, *n* (%)		
Variable	No	Yes	*P* value
Surgery on admission			
Yes	5 (13.9%)	31 (86.1%)	0.001^a^
Type of surgery			
Minimal	0 (0.0%)	4 (100.0%)	
NHSN	1(5.6%)	17 (94.4%)	1.000^b^
McCabe score			
Non-Fatal	62 (37.6%)	103 (62.4%)	
Rapidly Fatal	10 (52.6%)	9 (47.4%)	0.386^a^
Ultimately Fatal	3 (50.0%)	3 (50.0%)	
Urinary catheter			
Yes	6 (18.8%)	26 (81.3%)	
Peripheral Vascular Catheter			
Yes	38 (29.9%)	89 (70.1%)	<0.001^a^

NHSN, National Healthcare Safety Network surgery list defined in Annex IX of WHO protocol.^17^

a Chi-squared test was performed.

b Fisher’s exact test.

According to the multivariate analysis, the associations between surgery, peripheral vascular catheter and urinary catheter use with antibiotic use were not statistically significant on adjusting for other variables. Increasing age and malaria were associated with decreased adjusted odds of being on antibiotics (Table 6).

**Table 6.** Logistic regression to adjust variables for potential confounders (all facilities)

Variable	UAOR (95% CI)	AOR (95% CI)	*P* value
Patients on antibiotics			
Age in years	1.00 (0.98–1.01)	0.98 (0.96–1.00)	0.022
Gender			
Female	1	1	
Male	0.65 (0.35–1.21)	2.33 (0.88–6.20)	0.09
Surgery on Admission			
No	1	1	
Yes	5.1 (1.89–14.00)	3.2 (0.83–12.56)	0.092
Comorbidities			
McCabe Score			
Rapidly Fatal	1	1	
Non-Fatal	1.8 (0.71–4.79)	2.51 (0.60–10.49)	0.206
Ultimately Fatal	1.1 (0.18–6.97)	0.60 (0.04–9.83)	0.721
Use of catheter			
No	1	1	
Yes	3.5 (1.38–9.11)	4.26 (0.82–22.02)	0.084
Type of infection			
Malaria			
No	1	1	
Yes	0.4 (0.19–0.98)	0.33 (0.11–0.94)	0.038
Tuberculosis			
No	1		
Yes	0.2 (0.01–2.02)	–	–
HIV			
No	1		
Yes	1.5 (0.29–8.12)	–	–
Peripheral vascular catheter			
No	1	1	
Yes	3.5 (1.82–6.80)	2.31 (0.90–5.87)	0.08

UAOR, unadjusted odds ratio; AOR, adjusted odds ratio. Dashes represent variables that were dropped due to limited numbers and/or collinearity.

## Discussion

According to this study, the prevalence of antibiotic use is high relative to other studies, with many patients using two or more antibiotics, and antibiotics in *Watch* category in the WHO *AWaRe* classification.^7^^,^^23^ Furthermore, only a small fraction of patients had a sample taken for culture and susceptibility analyses before antibiotic use. The prevalence of patients on at least one antibiotic is comparable to findings from other surveys across Africa and the world. Previous studies in Ghana have recorded 61%–82% prevalence of antibiotic use in hospitals^11^^,^^13^ while other studies from around Africa have reported 50%–70.6% prevalence.^8^^,^^14^^,^^16^^,^^24^ Studies conducted outside Africa have reported higher prevalence of 77.6% (Pakistan)^10^ but mainly lower prevalence of 27%–50% in Europe and the United States.^14^^,^^15^^,^^25^^,^^26^ These studies employed standardized protocols and the differences in antibiotic use may be due to different living conditions, public education^27^ as well as differences in clinical practice. In our study, the differences in prevalence of antibiotic use may be attributable to the different level of hospital facilities in the study. The prevalence found in the tertiary facility UHS was similar to what was found in a similar facility in Ghana with prevalence of 51.4%.^12^ This may represent a comparable use of antibiotics across a similar hospital level. This finding supports the use of data from comparable institutions and from the specific facility for policy making. Subsequent interventions for improving antibiotic use will then have maximal impact in individual hospitals versus using generic data and interventions.

The majority of patients on antibiotics were on two antibiotics at the time of the study, which may be a key target for improvement. An example is a case observed in the study where a patient was on ceftriaxone, gentamicin and azithromycin for clinical sepsis. Such a combination is usually aimed at preventing or surmounting possible resistance. The conventional mantra in infectious disease management is to ‘hit hard and to hit early’, which may often include combination of antimicrobial agents for synergy.^28^ This strategy may be flawed, as, if executed poorly, it could lead to the exponential selection of resistance genes in microbes because competing organisms have been destroyed by the aggressive therapy.^29^ It is important therefore that therapy is guided by evidence from investigations.

However, only a small fraction of patients had a sample taken for culture and drug susceptibility analyses before antibiotic therapy was started. This is an important finding as directed therapy is essential in optimizing therapy with antibiotics compared with empirical therapy. Local antibiograms and antimicrobial guidelines are needed as interventions in the hospitals to direct empirical therapy in the face of limited resources. Amoxicillin, ciprofloxacin, ceftriaxone, cefuroxime and ampicillin were the most used antibiotics overall. This is comparable to the top three antibiotics prescribed worldwide.^14^

None of the facilities at the time of the study had patients on antibiotics classified in the *Reserve* category of the WHO *AWaRe* system, similar to another study in Ghana,^12^ although their availability was not assessed in the current study. However, a good number of antibiotics surveyed belonged to the *Watch* group with three of them among the most used agents overall, which was higher than findings in Finland (23%) but lower than in Iran (77.3%). Use of *Access* agents could be increased in Ghana (47%) when compared with that in Singapore (100%) for neonates.^23^ Interventions could be tailored at using this tool to direct antibiotic use in hospitals in low-resource settings.^23^^,^^30^ Since potentially only a small fraction of the antibiotics prescribed were culture- and susceptibility-directed therapy, using antibiotics in the *Watch* category as much could be potentially problematic. This can be addressed effectively and inexpensively with hospital-specific formularies with the *AWaRe* classification taken into consideration. Another intervention could be to increase use of *Access* agents and restrict the use of *Reserve* agents to require preauthorization from qualified persons to maintain their low use in the health facilities. Prescriber training and information posters could also be provided to support appropriate prescribing and compliance to rational antibiotic use strategies. Increasing age was associated with slightly decreased adjusted odds of being on an antibiotic. This may be attributed to paediatric patients having immature immune systems and so are at an understandably higher risk of infections requiring antibiotics compared with older individuals. Older persons have more developed immune systems which have evolved to provide protection through exposure to various foreign agents and pathogens from childhood to adulthood.^31^ A decline in old age is expected but this was not seen in the study as the overall age range for patients did not include geriatrics. Future PPSs could focus on paediatric and geriatric populations in Ghana for evidence to drive policymaking in these vulnerable groups.

A concurrent diagnosis of malaria was protective from receiving antibiotics in patients. Malaria is a febrile illness, endemic to Ghana,^32^ and with symptoms that could be mistaken for bacterial infections. The availability of cheap rapid diagnostic tests and the high suspicion for malaria in febrile illnesses in Ghana may explain the statistically significant protection. This finding may indicate that prescribers are not irrationally using antibiotics in malaria, which may be commendable.

There were statistically significant associations between having surgery on admission, having a urinary catheter, a peripheral vascular catheter (PVC) *in situ* and being on an antibiotic in univariate analyses. This finding may be due to their invasive nature, but could be confounded by the severity of illness in these patients requiring such devices or procedures. Catheter-related urinary tract infections are one of the most common nosocomial infections and a common complication of urinary catheters.^33–35^ Widespread PVC use (estimated to be as high as in up to 80% of inpatients) may contribute to the association with antibiotic use.^36^ PVC use is associated with severe nosocomial infections and PVC-related nosocomial infections provide a target for reducing infections and antibiotic use.^37^^,^^38^ Adequate infection prevention and control when placing such devices, in addition to their maintenance, may help prevent nosocomial infections related to their use. This may especially be important in APH, where PVC use was significantly associated with antibiotic use while having surgery on admission was not (Table S1). In UHS, surgery and urinary catheter use were significantly associated with antibiotic use, whereas no associations were observed in EGH (Tables S2 and S3).

Antibiotic consumption data are essential in the fight against AMR as they provide an indication of prescribers’ behaviours and are an important metric for improving antibiotic use. This study represents the successful use of the WHO tool^17^ in surveying antibiotic use in hospitals. It will be especially useful in low-resource settings (as found in low- and middle-income countries) as it is not resource intensive. Our study is limited by the fact that a single PPS may be inadequate to provide information on prescription patterns and trends in antibiotic use in facilities. Subsequent PPS may be conducted during the same period of the initial PPS to account for possible trends in antibiotic use. A strength of our study includes the use of the standardized WHO tool to collect data and the use of multiple centres to collect data.

### Conclusions

The prevalence of antibiotic consumption in the hospitals was low relative to reports from some studies in Ghana, but high relative to similar studies from some high-income countries. Most of the antibiotic therapy was empirical and not guided by culture and drug susceptibility testing. The most commonly prescribed antibiotics were penicillins, cephalosporin and fluoroquinolones, with a great proportion being those in the WHO *Watch* category. Increasing age and patients diagnosed with malaria had lower odds of being prescribed antibiotics. There is the need for pragmatic stewardship initiatives in these hospitals to control the prescribing and consumption of antimicrobials. An example may be the application of the WHO *AWaRe* classification for the selection of antibiotics, as well as increased use of culture and antimicrobial susceptibility data to guide infectious disease therapy.

## Supplementary Material

dlab008_Supplementary_Data
