# Supplementary material for: Point prevalence survey of antibiotic consumption across three hospitals in Ghana
Source: JAC Antimicrob Resist. 2021 Feb 8;3(1):dlab008. doi: 10.1093/jacamr/dlab008 (PMC8210176; doi:10.1093/jacamr/dlab008)
Supplement: dlab008_Supplementary_Data [file dlab008_supplementary_data.docx]

**Supplementary data**

Table S1. Patient variables and association with antibiotic use in APH (n=101)

| Type of facility | Variable | Patients on Antibiotics | | p value |
| --- | --- | --- | --- | --- |
|  |  | **No (%)** | **Yes (%)** |  |
| APH | Surgery on admission |  |  |  |
|  | No | 24(35.3%) | 44(64.7%) | 0.161 |
|  | Yes | 4(19.1%) | 17(81.0%) |  |
|  | Urinary Catheter |  |  |  |
|  | No | 31(39.2%) | 48(60.8%) | **0.041** |
|  | Yes | 2(12.5%) | 14(87.5%) |  |
|  | Peripheral Vascular Catheter |  |  |  |
|  | No | 16(64.0%) | 9(36.0%) | **<0.001** |
|  | Yes | 16(22.2%) | 56(77.8%) |  |

Table S2. Patient variables and association with antibiotic use in UHS (n=xx)

| Facility | Variable | Patients on Antibiotics | | p value |
| --- | --- | --- | --- | --- |
|  |  | **No (%)** | **Yes (%)** |  |
| UHS | Surgery on admission |  |  |  |
|  | No | 17(54.8%) | 14(45.2%) | **0.008** |
|  | Yes | 1(9.1%) | 10(90.9%) |  |
|  | Urinary Catheter |  |  |  |
|  | No | 17(54.8%) | 14(45.2%) | **0.036** |
|  | Yes | 2(18.2%0 | 9(81.8%) |  |
|  | Peripheral Vascular Catheter |  |  |  |
|  | No | 9(50.0%) | 9(50.0%) | 0.486 |
|  | Yes | 9(39.1%) | 14(60.9%) |  |

Table S3. Patient variables and association with antibiotic use in EGH (n=xx)

| Type of facility | Variable | Patients on Antibiotics | | p value |
| --- | --- | --- | --- | --- |
|  |  | **No (%)** | **Yes (%)** |  |
| EGH | Surgery on admission |  |  |  |
|  | No | 22(55.0%) | 18(45.0%) | 0.108* |
|  | Yes | 0(0.0%) | 4(100.0%) |  |
|  | Urinary Catheter |  |  |  |
|  | No | 19(48.7%) | 20(51.3%) | 0.713 |
|  | Yes | 2(40.0%) | 3(60.0%) |  |
|  | Peripheral Vascular Catheter |  |  |  |
|  | No | 8(66.7%) | 4(33.3%) | 0.124 |
|  | Yes | 13(40.6%) | 19(59.4%) |  |

***Chi squared test was performed in each case. *Fisher’s exact test. p value < 0.05 is considered significant***
